# Supplementary material for: A mechanism to prevent production of reactive oxygen species by Escherichia coli respiratory complex I
Source: Nat Commun. 2019 Jun 11;10:2551. doi: 10.1038/s41467-019-10429-0 (PMC6560083; doi:10.1038/s41467-019-10429-0)
Supplement: Supplementary file 1 — Supplementary Information [file 41467_2019_10429_MOESM1_ESM.pdf]

# Supplementary Information

## A mechanism to prevent production of reactive oxygen species by *Escherichia coli* respiratory complex I

Marius Schulte[1], Klaudia Frick[1], Emmanuel Gnandt[1], Sascha. Jurkovic[1], Sabrina Burschel[1], Ramona Labatzke[1], Karoline Aierstock[1,2], Dennis Fiegen[2], Daniel Wohlwend[1], Stefan Gerhardt[1], Oliver Einsle[1,3], Thorsten Friedrich[1]

[1]Institut für Biochemie, Albert-Ludwigs-Universität Freiburg, Albertstr. 21, 79104 Freiburg, Germany.

[2]Boehringer Ingelheim Pharma GmbH & Co. KG, Lead Identification and Optimization Sup., 88397 Biberach, Germany.

[3]BIOSS Centre for Biological Signalling Studies, Schänzlestrasse 1, 79104 Freiburg, Germany.

**This PDF file includes:**

**Supplementary Figure 1**: Sequence alignment of subunits NuoE (A) and NuoF (B) from *A. aeolicus*, *T. thermophilus* and *E. coli.*

**Supplementary Figure 2**: Backbone superposition of *A. aeolicus* NuoEF and *T. thermophilus* Nqo1 and Nqo2.

**Supplementary Figure 3**: Structure of the *A. aeolicus* G129$^{E}$ variants in the reduced and oxidized state.

**Supplementary Figure 4**: Conservation of the residues establishing the flipping peptide bond.

**Supplementary Figure 5**: Characterization of the G135$^{E}$ variant preparations.

**Supplementary Figure 6**: Thermal stability of the flavin site in the parental protein and the G135S$^{E}$ and G135D$^{E}$ variants of complex I.

**Supplementary Figure 7**: Example of electron density of the NADH oxidation site with bound NADH.

**Supplementary Table 1**: Data collection and refinement statistics.

**Supplementary Table 2**: Interactions of NADH with NuoEF.

**Supplementary Table 3**: Interactions of $NAD^{+}$ with NuoEF.

**Supplementary Table 4**: Preparation of the *A.aeolicus* G129S$^{E}$ and G129D$^{E}$ and the *E. coli* G135S$^{E}$ and G135D$^{E}$ variants.

**Supplementary Table 5**: Mutagenic primer to construct the *A. aeolicus* G129S$^{E}$, G129D$^{E}$ and S96M$^{F}$ and the *E. coli* G135S$^{E}$ and G135D$^{E}$ variants.

**Supplementary Note** on binding of $NAD^{+}$ and NADH to *A. aeolicus* NuoEF.

**a)**

```
A.ae    ------MFKTEFEFPEELKTKLQEHINYFPK--KRQAILLCLHEIQNYYGYIPPESLKPL 52
T.th    -----------MGFFDDKQDFLEETFAKYPPEGRRAAIMPLLRRVQQEEGWIRPERIEEI 49
E.co    MHENQQPQTEAFELSAAEREAIEHEMHHYED--PRAASIEALKIVQKQRGWVPDGAIHAI 58
                   : :    :  ::. :  :     * * :  *: :*:  *::    :. :

A.ae    ADMLELPLNHVEGVVAFYDMFDREDKAKYRIRVCVSIVCHLMGTNKLLKALENILGIKPG 112
T.th    ARLVGTTPTEVMGVASFYSYYQFVPTGKYHLQVCATLSCKLAGAEELWDYLTETLGIGPG 109
E.co    ADVLGIPASDVEGVATFYSQIFRQPVGRHVIRYCDSVVCHINGYQGIQAALEKKLNIKPG 118
        * ::  . ..* **.:**.       .:: :: * :: *:: * : :   * : *.* **

A.ae    EVTPDGKFKIVPVQCLGACSEAPVFMVNDDEY-KFESEVQLNEILSRYT----------- 160
T.th    EVTPDGLFSVQKVECLGSCHTAPVIQVNDEPYVECVTRARLEALLAGLRAGKRLEEIELP 169
E.co    QTTFDGRFTLLPTCCLGNCDKGPNMMIDEDTH-AHLTPEAIPELLERYK----------- 166
        :.* ** *.:  . *** *  .* : :::: :    :   :  :*

T.th    GKCGHHVHEVEV 181
```

**b)**

```
A.ae       MRSYPAIPRIYAETTLNMLLKRAKKPRVHSIDEYLKDGGYQALEKALN-MSPEEIIDWVD 59
T.th       -MTGPILSGLDPRFER-TLYAHVGKEGSWTLDYYLRHGGYETAKRVLKEKTPDEVIEEVK 58
E.co       --MKNIIR--TPETHPLTWRLRDDKQPVW-LDEYRSKNGYEGARKALTGLSPDEIVNQVK 55
                :    ..        :  *     :* *  ..**:  .:.*.  :*:*::: *.

A.ae       KSTLRGRGGAGFPTGKKWKFAVQNPG--PRYFICNADESEPGTFKDRIIIERDPHLLIEG 117
T.th       RSGLRGRGGAGFPTGLKWSFMPKDDGK-QHYLICNADESEPGSFKDRYILEDVPHLLIEG 117
E.co       DAGLKGRGGAGFSTGLKWSLMPKDESMNIRYLLCNADEMEPGTYKDRLLMEQLPHLLVEG 115
            : *:*******.** **.:  :: .   :*::***** ***::*** ::*  ****:**

A.ae       IIISSYAIGANEAYIYIRGEYPAGYYILRDAIEEAKKKGFLGKNILGSGFDLEIYVARGA 177
T.th       MILAGYAIRATVGYIYVRGEYRRAADRLEQAIKEARARGYLGKNLFGTDFSFDLHVHRGA 177
E.co       MLISAFALKAYRGYIFLRGEYIEAAVNLRRAIAEATEAGLLGKNIMGTGFDFELFVHTGA 175
           ::::.:*: *  .**::****  .   *. ** **   * ****::*:.*.:::.*  **

A.ae       GAYICGEETALIESLEGKRGHPRLKPPYPVQKGLWGKPTVVNNVETIANVPFIISMGWEE 237
T.th       GAYICGEETALMNSLEGLRANPRLKPPFPAQSGLWGKPTTINNVETLASVVPIMERGADW 237
E.co       GRYICGEETALINSLEGRRANPRSKPPFPATSGAWGKPTCVNNVETLCNVPAILANGVEW 235
           * *********::**** *.:** ***:*. .* ***** :*****:..*  *:  * :

A.ae       YRYIGPSDYAGPKLFPVSGKVKKPGVYELPMNTTLREVIFKYAGGTLGNKKVKAVFSGAL 297
T.th       FAQMGTEQSKGMKLYQISGPVKRPGVYELPMGTTFRELIYEWAGGPLEP--IQAIIPGGS 295
E.co       YQNISKSKDAGTKLMGFSGRVKNPGLWELPFGTTAREILEDYAGGMRDGLKFKAWQPGGA 295
           :  :. ..   * **  .** **.**::***:.** **:: .:***       .:*  .*.
A.ae       DC----FSSEELDIPMDYSPLGFG----GTGTVIVLTEEDDIVEAALKIAEFYEHETCGQ 349
T.th       STPPLPFTEEVLDTPMSYEHLQAKGSMLGTGGVILIPERVSMVDAMWNLTRFYAHESCGK 355
E.co       GTD--FLTEAHLDLPMEFESIGKAGSRLGTALAMAVDHEINMVSLVRNLEEFFARESCGW 353
           .     ::.  ** **.:. :       **. .: : .. .:*.   :: .*: :*:**

A.ae       CTPCRVGCY-EQANLLEKIYKGEATEQDWEGFDFVNRNIQP-TSICGLGAVAGRLIRQTL 407
T.th       CTPCREGVAGFMVNLFAKIGTGQGEEKDVENLEALLPLIEG-RSFCPLADAAVWPVKGSL 414
E.co       CTPCRDGLP-WSVKILRALERGEGQPGDIETLEQLCRFLGPGKTFCAHAPGAVEPLQSAI 412
           ***** *     .:::  :  *:.   * * :: :   :    ::*  .  *   :: ::

A.ae       EKFPEEWEKYRKKSASLPL-------------- 426
T.th       RHFKDQYLALAREKRPVPRPSLWR--------- 438
E.co       KYFREEFEAGIKQPFSNTHLINGIQPNLLKERW 445
           . * :::    ::  .
```

**Supplementary Figure 1 | Sequence alignment of subunits NuoE (A) and NuoF (B) from *A. aeolicus* (A.ae), *T. thermophilus* (T.th) and *E. coli* (E.co).** **a**) Alignment of NuoF orthologes. The sequence identity between *A. aeolicus* and *T. thermophilus* is 32%, that between *E. coli* and *A. aeolicus* 31% and that between *E. coli* and *T. thermophilus* 24%. **b**) Alignment of NuoE orthologes. The sequence identity between *A. aeolicus* and *T. thermophilus* is 45%, that between *E. coli* and *A. aeolicus* 43% and that between *E. coli* and *T. thermophilus* 45%.

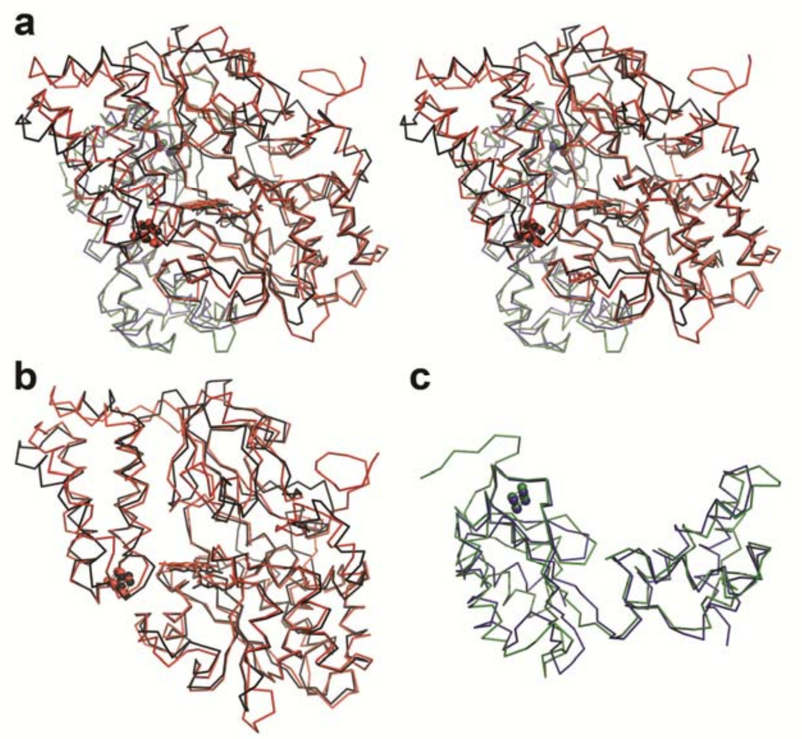


**Supplementary Figure 2 | Backbone superposition of *A. aeolicus* NuoEF and *T. thermophilus* Nqo1 and Nqo2. a**) Stereo representation of a superposition of $C_\alpha$-traces for *A. aeolicus* NuoEF and *T. thermophilus* Nqo1and 2. For *A. aeolicus*, NuoF is shown in black and NuoE in blue, for *T. thermophilus* Nqo1 is shown in red and Nqo2 in grey. **b**) Superposition of *A. aeolicus* NuoF with *T. thermophilus* Nqo1. In spite of a slight difference in the positioning of FMN the overall structure of the two orthologs is very similar (rmsd: 1.2 Å for all atoms). **c**) Overlay of *A. aeolicus* NuoE with *T. thermophilus* Nqo2 highlighting the two-domain architecture. The rmsd is 1.8 Å for all atoms.

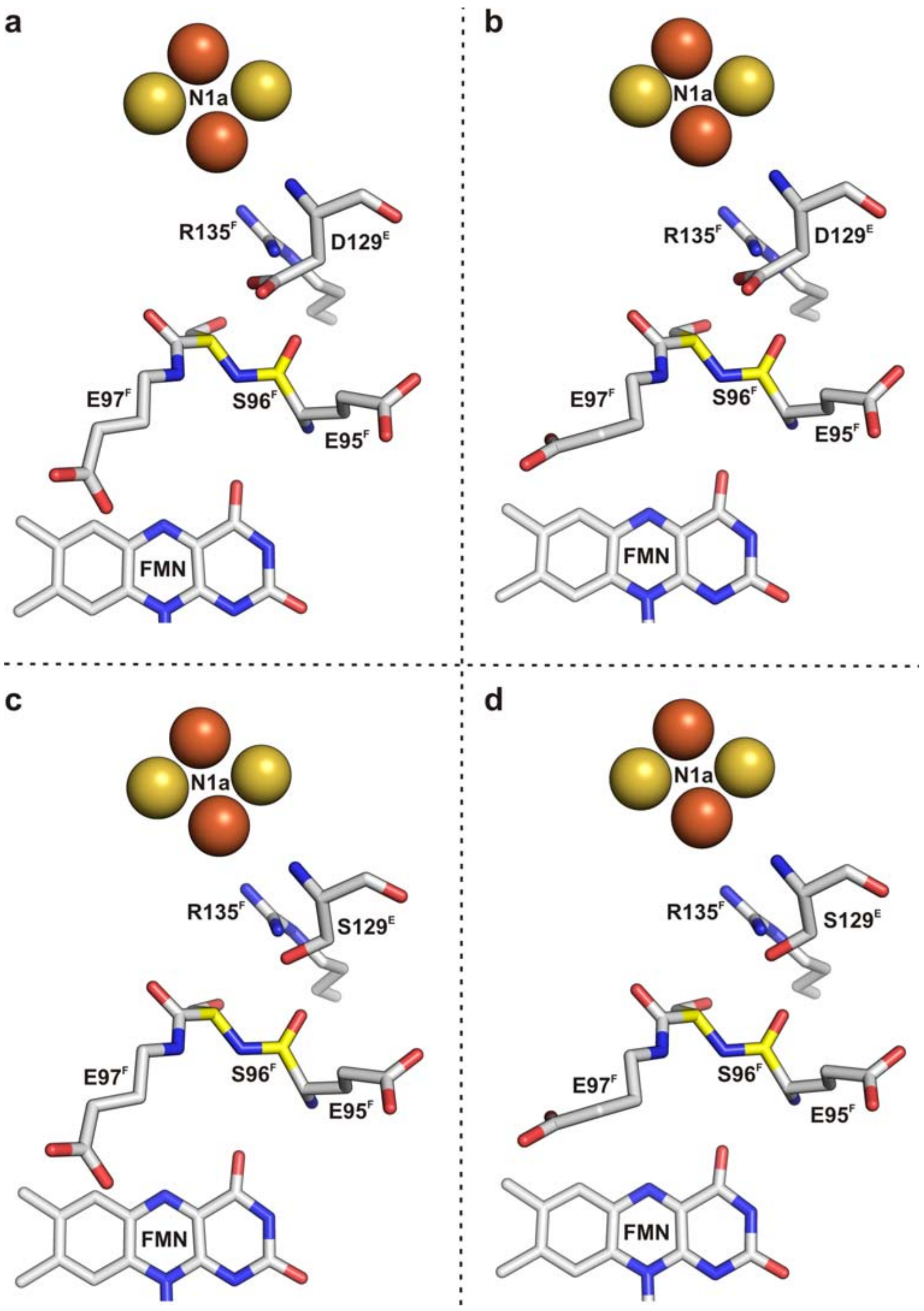


**Supplementary Figure 3 |** Structure of the *A. aeolicus* G129$^{E}$ variants in the reduced and oxidized state. **a)** Structure of the G129D$^{E}$ variant reduced by NADH and dithionite. **b)** Structure of the G129D$^{E}$ variant in the oxidized state. **c)** Structure of the G129S$^{E}$ variant reduced by NADH and dithionite. **d)** Structure of the G129S$^{E}$ variant in the oxidized state. The position of the Fe-S cluster N1a and the FMN isoalloxazine ring is given. The position of the flipping peptide bond indicated by the yellow colored carbon atoms does not change in dependence of the redox state of NuoEF.

**a)**

```
Bovine           KYLVVNADEG EPGTCKDREI IRHDPH
Mouse            KYLVVNADEG EPGTCKDREI MRHDPH
Rat              KYLVVNADEG EPGTCKDREI MRHDPH
Human            KYLVVNADEG EPGTCKDREI IRHDPH
Y. lipolytica    RYLVVNADEG EPGTCKDREI MRKDPH
Paracoccus       SYLVINADES EPATCKDREI MRHDPH
E. coli          RYLLCNADEM EPGTYKDRLL MEQLPH
T. thermophilus  HYLICNADES EPGSFKDRYI LEDVPH
A. aeolicus      RYFICNADES EPGTFKDRII IERDPH
Consensus         *    ****  **    ***         **
```

**b)**

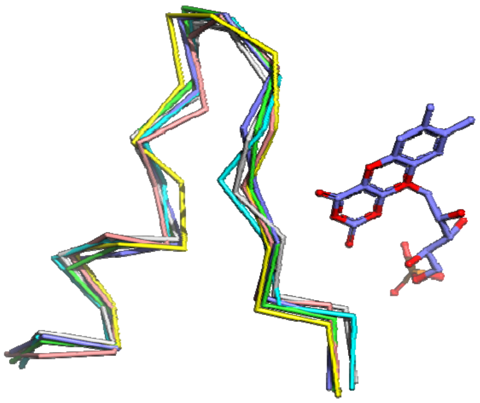

**c)**

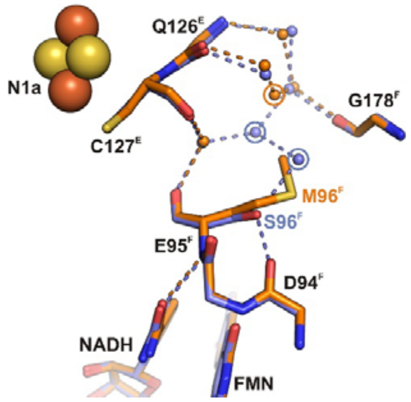


**Supplementary Figure 4 |** Conservation of the residues establishing the flipping peptide bond. **a)** Sequence alignment of NuoF from different organisms in the region containing the flipping peptide bond. The sequence from R87 to H112 (*A. aeolicus* numbering) is shown. The not conserved position 96 is marked in yellow. **b)** Superposition of the NuoF protein backbone of bovine, human, mouse and rat mitochondrial complex I and of the *T. thermophilus* and *A. aeolicus* homologues from C91 to R109 (*A. aeolicus* numbering). The position of the FMN is shown for orientation. The rmsd between the individual structures ranges from 0.42 to 1.36 Å, the mean value is 0.7 Å. Thus, the structures are considered to be identical. **c)** Structure of the reduced NuoEF (blue) and S96M$^F$ variant (orange). The water molecules displaced by the methonine residue are circled in blue, the water molecule not present in the original protein is circled in orange. The position of the other amino acid side chain did not change.

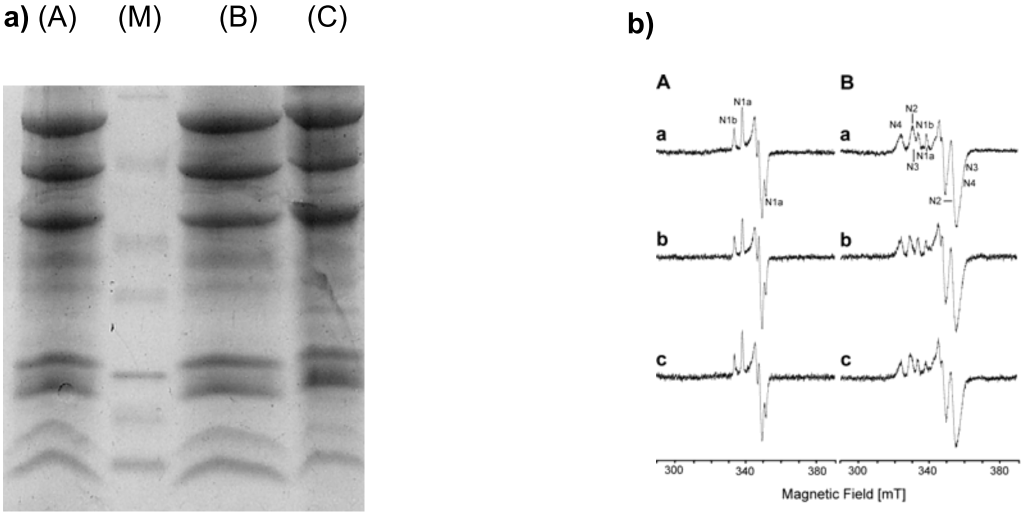


**Supplementary Figure 5 | Characterization of the G135$^E$ variant preparations. a)** SDS-PAGE of the preparations from the parental strain (A), the G135S$^E$ (B) and the G135D$^E$ mutant strain (C). Each lane was loaded with 50 µg protein. The gel was stained with Coomassie brilliant blue leading to a weaker staining of the hydrophobic proteins. Lane (M) shows the pattern of the Unstained Protein Molecular Weight Marker. **b)** EPR spectra of the preparation of the parental protein (a), the G135S$^E$ variant (b), and the G135D$^E$ variant (c) at 40 K and 2 mW (A) and 13 K and 5 mW (B). The samples were reduced with a 1000-fold excess NADH under anoxic conditions. The absorbances of the individual clusters are indicated in the spectra of the parental protein. Source data are provided as a Source Data file.

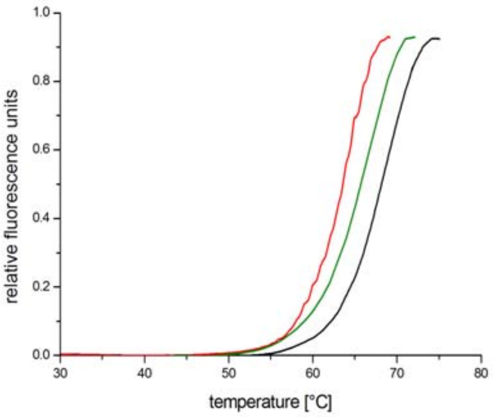


**Supplementary Figure 6 | Thermal stability of the flavin site in the parental protein (black) and the G135S$^E$ (green) and G135D$^E$ (red) variants of complex I.** The fluorescence of free FMN was recorded with a real-time PCR machine while the temperature was constantly increased. The curves show the average data points of three measurements. The standard error per data point was less than 1%. The calculated 'melting points' were 68.6 ± 0.1°C for the parental complex, 64.0 ± 0.1°C for the G135S$^E$ variant and 63.2 ± 0.1°C for the G135D$^E$ variant. Assay conditions: 1 mg/ml protein in 50 mM MES/NaOH, 50 mM NaCl, 0.1% (w/v) dodecyl-maltoside, pH 6.0. Source data are provided as a Source Data file.

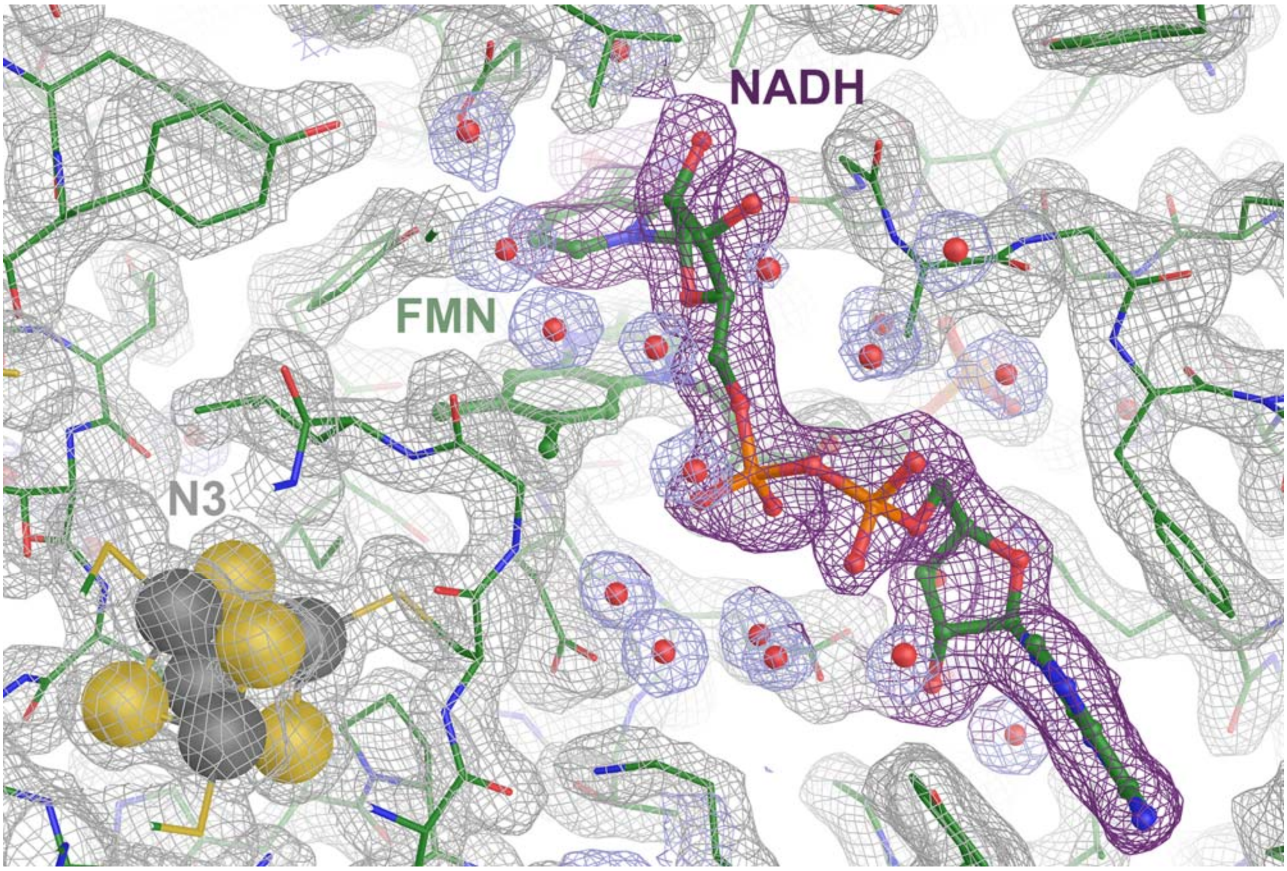


**Supplementary Figure 7 | Example of electron density of the NADH oxidation site with bound NADH.** $2F_o$-$F_c$ electron density is contoured at 1 σ. Final atomic models are shown in the element colors. Sulfur of the Fe-S clusters is shown in yellow, iron grey. Water is shown in red. The electron density of NADH is shown in purple.

## Supplementary Table 1 | Data collection and refinement statistics.

| Data Set | Oxidized (Ox) | Ox plus NADH (reduced) | Ox plus $NAD^+$ (oxidized) | Ox plus Dithionite (reduced) |
|---|---|---|---|---|
| space group | $P2_12_12_1$ | $P2_12_12_1$ | $P2_12_12_1$ | $P2_12_12_1$ |
| wavelength [Å] | 1.65308 | 1.00000 | 1.54179 | 1.54179 |
| cell constants *a*, *b*, *c* [Å] | 63.2, 116.4, 189.4 | 63.3, 115.8, 189.8 | 63.4, 115.8, 189.6 | 59.7, 108.1, 188.2 |
| α, β, γ [°] | 90.0, 90.0, 90.0 | 90.0, 90.0, 90.0 | 90.0, 90.0, 90.0 | 90.0, 91.7, 90.0 |
| resolution limits [Å] | 99.2 – 1.95 (1.98 – 1.95) | 49.44 – 1.78 (1.81 – 1.78) | 73.35 – 2.04 (2.15 – 2.04) | 188.2 – 2.06 (2.18 – 2.06) |
| completeness (%) | 99.9 (97.7) | 100.0 (100.0) | 97.6 (95.6) | 86.7 (71.1) |
| unique reflections | 102,591 (4,912) | 134,290 (6,611) | 88,168 (12,408) | 65,727 (7,715) |
| multiplicity (%) | 15.9 (10.0) | 13.7 (13.8) | 7.4 (7.5) | 4.9 (3.1) |
| $R_{merge}$ [a)] | 0.162 (1.561) | 0.190 (1.436) | 0.123 (0.904) | 0.110 (0.390) |
| $R_{p.i.m.}$ | 0.044 (0.53) | 0.077 (0.577) | 0.065 (0.486) | 0.060 (0.242) |
| mean I/ σ(I) | 12.9 (1.5) | 7.2 (1.6) | 14.4 (2.3) | 9.3 (2.7) |
| $CC_{1/2}$ | 0.998 (0.662) | 0.995 (0.813) | 0.998 (0.780) | 0.994 (0.795) |
| **refinement statistics** | | | | |
| $R_{cryst}$ [b)] | 0.169 | 0.180 | 0.173 | 0.200 |
| $R_{free}$ (%) | 0.195 | 0.207 | 0.203 | 0.242 |
| non-hydrogen atoms | 9084 | 10,355 | 9073 | 9054 |
| solvent molecules | 693 | 961 | 530 | 127 |
| **r.m.s. deviations from ideal values** | | | | |
| bond lengths (Å) | 0.009 | 0.004 | 0.008 | 0.010 |
| bond angles (º) | 0.93 | 1.22 | 0.93 | 1.08 |
| **average *B* values ($Å^2$)** | | | | |
| protein main chain atoms | 29.1 | 30.0 | 32.2 | 29.5 |
| protein all atoms | 32.8 | 31.0 | 35.8 | 33.2 |
| FMN | 20.6 | 20.3 | 22.0 | 21.6 |
| NAD | - | 26.1 | 43.6 | - |
| FES | 22.1 | 22.6 | 25.0 | 24.7 |
| SF4 | 23.6 | 21.1 | 29.7 | 25.1 |
| solvent | 34.8 | 40.6 | 37.1 | 23.4 |
| Wilson plot | 25.1 | 29.3 | 30.0 | 24.2 |

[a] $R_{merge} = \Sigma_{hkl} [(\Sigma_i |I_i - \langle I \rangle|) / \Sigma_i I_i]$

[b] $R_{cryst} = \Sigma_{hkl} ||F_{obs}| - |F_{calc}|| / \Sigma_{hkl} |F_{obs}|$

$R_{free}$ is the cross-validation *R* value for a test set of 5 % of unique reflections.

## Supplementary Table 1 | Data collection and refinement statistics (continued).

| Data Set | Reduced (Dithionite + NADH) | Reduced (Dithionite + NAD$^+$) | G129D oxidized plus NAD$^+$ | G129D reduced (Dithionite + NADH) |
|---|---|---|---|---|
| space group | $P2_12_12_1$ | P212121 | $P2_1$ | $P2_12_12_1$ |
| wavelength [Å] | 1.00000 | 1.54179 | 1.54179 | 1.54187 |
| cell constants *a*, *b*, *c* [Å] | 63.6, 116.4, 189.5 | 63.6, 116.0, 189.9 | 92.7, 63.3, 124.2 | 63.3, 116.2, 189.8 |
| α, β, γ [°] | 90.0, 90.0, 90.0 | 90.0, 90.0, 90.0 | 90.0, 107.2, 90.0 | 90.0, 90.0, 90.0 |
| resolution limits [Å] | 189.45 – 1.90 (1.93 – 1.90) | 116.0 – 2.38 (2.51 – 2.38) | 118.7 – 1.80 (1.83 – 1.80) | 26.40 – 2.10 (2.14 – 2.10) |
| completeness (%) | 98.7 (83.7) | 100.0 (100.0) | 90.1 (92.8) | 99.8 (99.5) |
| unique reflections | 110,071 (4,524) | 57,523 (8,284) | 114,854 (5,845) | 82,411 (4,411) |
| multiplicity | 7.0 (5.5) | 6.4 (6.4) | 2.7 (2.5) | 6.4 (5.6) |
| $R_{merge}$ [a)] | 0.143 (0.743) | 0.105 (0.757) | 0.035 (0.508) | 0.185 (0.835) |
| $R_{p.i.m.}$ | 0.086 (0.499) | 0.051 (0.351) | 0.037 (0.509) | 0.118 (0.562) |
| mean I/ σ(I) | 9.5 (2.3) | 13.2 (2.2) | 14.7 (1.4) | 6.5 (1.6) |
| $CC_{1/2}$ | 0.995 (0.577) | 0.998 (0.778) | 0.998 (0.602) | 0.993 (0.658) |
| **refinement statistics** | | | | |
| $R_{cryst}$ [b)] | 0.215 | 0.206 | 0.177 | 0.191 |
| $R_{free}$ (%) | 0.240 | 0.238 | 0.197 | 0.231 |
| non-hydrogen atoms | 9101 | 9095 | 9090 | 10,337 |
| solvent molecules | 670 | 248 | 497 | 961 |
| **r.m.s. deviations from ideal values** | | | | |
| bond lengths (Å) | 0.009 | 0.010 | 0.010 | 0.005 |
| bond angles (º) | 1.00 | 1.04 | 0.95 | 1.30 |
| **average *B* values (Å$^2$)** | | | | |
| protein main chain atoms | 26.3 | 47.3 | 30.8 | 25.4 |
| protein all atoms | 29.6 | 50.9 | 34.1 | 26.3 |
| FMN | 15.6 | 37.6 | 21.1 | 15.5 |
| NAD | 22.5 | 45.5 | 43.8 | 20.7 |
| FES | 20.9 | 38.8 | 24.8 | 16.3 |
| SF4 | 22.2 | 48.2 | 26.6 | 15.8 |
| solvent | 30.1 | 39.7 | 36.6 | 32.7 |
| Wilson plot | 21.1 | 45.7 | 27.3 | 24.7 |

[a] $R_{merge} = \Sigma_{hkl} [(\Sigma_i |I_i - \langle I \rangle|) / \Sigma_i I_i]$

[b] $R_{cryst} = \Sigma_{hkl} ||F_{obs}| - |F_{calc}|| / \Sigma_{hkl} |F_{obs}|$

$R_{free}$ is the cross-validation *R* value for a test set of 5 % of unique reflections.

## Supplementary Table S1 | Data collection and refinement statistics (continued).

| Data Set | G129S oxidized | G129S reduced (Dithionite + NADH) | S96M oxidized | S96M reduced (Dithionite + NADH) |
|---|---|---|---|---|
| space group | $P2_12_12_1$ | $P2_12_12_1$ | $P2_12_12_1$ | $P2_12_12_1$ |
| wavelength [Å] | 1.54179 | 1.00000 | 1.54187 | 1.54187 |
| cell constants *a*, *b*, *c* [Å] | 62.6, 115.8, 189.9 | 63.6, 116.3, 190.3 | 63.4, 114.6, 187.9 | 63.4, 116.0, 190.0 |
| α, β, γ [°] | 90.0, 90.0, 90.0 | 90.0, 90.0, 90.0 | 90.0, 90.0, 90.0 | 90.0, 90.0, 90.0 |
| resolution limits [Å] | 98.9 – 2.10 (2.14 – 2.10) | 48.2 – 1.83 (1.86 – 1.83) | 38.2 – 3.22 (3.48 – 3.22) | 21.3 – 1.99 (2.02 – 1.99) |
| completeness (%) | 99.9 (99.9) | 99.6 (99.5) | 99.8 (100.0) | 99.8 (99.3) |
| unique reflections | 81,432 (4,421) | 124,614 (6,107) | 22,909 (4,655) | 96,646 (4,699) |
| multiplicity (%) | 6.4 (6.4) | 13.7 (14.1) | 6.7 (6.8) | 5.8 (5.0) |
| $R_{merge}$ [a)] | 0.128 (0.987) | 0.148 (1.707) | 0.248 (0.822) | 0.162 (0.776) |
| $R_{p.i.m.}$ | 0.061 (0.463) | 0.059 (0.681) | 0.151 (0.505) | 0.108 (0.528) |
| mean I/ σ(I) | 10.5 (1.8) | 11.8 (1.7) | 6.8 (2.0) | 6.9 (1.6) |
| $CC_{1/2}$ | 0.998 (0.698) | 0.999 (0.761) | 0.991 (0.795) | 0.992 (0.699) |
| **refinement statistics** | | | | |
| $R_{cryst}$ [b)] | 0.179 | 0.172 | 0.208 | 0.188 |
| $R_{free}$ (%) | 0.218 | 0.197 | 0.257 | 0.219 |
| non-hydrogen atoms | 9097 | 10,377 | 9,380 | 10,338 |
| solvent molecules | 544 | 981 | 113 | 959 |
| **r.m.s. deviations from ideal values** | | | | |
| bond lengths (Å) | 0.009 | 0.004 | 0.005 | 0.005 |
| bond angles (°) | 0.99 | 1.22 | 1.32 | 1.27 |
| **average *B* values (Å²)** | | | | |
| protein main chain atoms | 36.0 | 28.9 | 64.2 | 22.2 |
| protein all atoms | 39.3 | 30.0 | 66.0 | 23.0 |
| FMN | 26.9 | 19.3 | 67.2 | 13.4 |
| NAD | - | 24.1 | - | 18.1 |
| FES | 26.0 | 21.1 | 35.4 | 14.4 |
| SF4 | 27.5 | 20.6 | 83.6 | 12.9 |
| solvent | 34.8 | 39.6 | 32.2 | 30.3 |
| Wilson plot | 29.7 | 28.4 | 60.3 | 21.7 |

[a] $R_{merge} = \Sigma_{hkl} [(\Sigma_i |I_i - \langle I \rangle|) / \Sigma_i I_i]$

[b] $R_{cryst} = \Sigma_{hkl} ||F_{obs}| - |F_{calc}|| / \Sigma_{hkl} |F_{obs}|$

$R_{free}$ is the cross-validation *R* value for a test set of 5 % of unique reflections.

**Supplementary Table 2 | Interactions of NADH with reduced NuoEF.**

| **First coordination sphere** | | | **Second coordination sphere** | | |
|---|---|---|---|---|---|
| **Atom NADH** | **Residue Atom** | **Length [Å]** | **Residue Atom** | **Residue Atom** | **Length [Å]** |
| AO3' | Lys76$^{F}$ NZ | $H_B$ 3.0 | Lys76$^{F}$ N | Pro72$^{F}$ O | $H_B$ 3.3 |
| | | | Lys76$^{F}$ NZ | Val218$^{F}$ O | $H_B$ 2.7 |
| | | | Lys76$^{F}$ O | Ala80$^{F}$ N | $H_B$ 3.4 |
| | | | Lys76$^{F}$ NZ | FMN O2P | $H_B$ 2.7 |
| AO2' | Glu185$^{F}$ OE1 | $H_B$ 3.3 | Glu185$^{F}$ OE2 | HOH 540 | $H_B$ 2.7 |
| AO2' | Glu185$^{F}$ OE2 | $H_B$ 2.5 | Glu185$^{F}$ O | Ile189$^{F}$ N | $H_B$ 3.1 |
| AO3' | Glu185$^{F}$ OE1 | $H_B$ 2.6 | Glu185$^{F}$ N | FMN O2' | $H_B$ 3.0 |
| | | | Glu185$^{F}$ OE1 | HOH 103 | $H_B$ 3.2 |
| AO2' | HOH 528 | $H_B$ 2.7 | HOH 528 | HOH 103 | $H_B$ 2.9 |
| | | | HOH 528 | AN3 | $H_B$ 3.0 |
| NO2' | HOH 545 | $H_B$ 2.7 | HOH 545 | Glu97$^{F}$ OE2 | $H_B$ 2.9 |
| | | | HOH 545 | HOH 541 | $H_B$ 3.2 |
| NO7 | Glu97$^{F}$ N | $H_B$ 3.0 | Glu97$^{F}$ O | Thr100$^{F}$ N | $H_B$ 3.0 |
| | | | Glu97$^{F}$ O | Thr100$^{F}$ OG1 | $H_B$ 3.2 |
| | | | Glu97$^{F}$ OE2 | Tyr180$^{F}$ OH | $H_B$ 2.7 |
| | | | Glu97$^{F}$ OE1 | Ala296$^{F}$ N | $H_B$ 2.9 |
| | | | Glu97$^{F}$ OE2 | HOH 545 | $H_B$ 2.9 |
| | | | Glu97$^{F}$ O | HOH 32 | $H_B$ 3.1 |
| | | | Glu97$^{F}$ OE1 | HOH 82 | $H_B$ 2.7 |
| | | | Glu97$^{F}$ OE2 | HOH 82 | $H_B$ 3.3 |
| NN7 | Gly67F O | $H_B$ 3.2 | | | |
| NN7 | HOH 526 | $H_B$ 2.9 | HOH 526 | Glu95$^{F}$ OE2 | $H_B$ 2.7 |
| | | | HOH 526 | Asp103$^{F}$ OD2 | $H_B$ 2.8 |
| NO1 | HOH 534 | $H_B$ 2.7 | HOH 534 | Gly394$^{F}$ N | $H_B$ 3.1 |
| | | | HOH 534 | Glu184$^{F}$ OE2 | $H_B$ 2.7 |
| | | | HOH 534 | HOH 514 | $H_B$ 2.7 |
| NO1 | HOH 567 | $H_B$ 2.7 | HOH 567 | HOH 261 | $H_B$ 2.7 |
| NO2 | FMN O2' | $H_B$ 3.1 | | | |
| NO2 | FMN O3' | $H_B$ 2.7 | | | |
| NO2 | HOH 540 | $H_B$ 2.7 | HOH 540 | Glu185$^{F}$ OE2 | $H_B$ 2.7 |
| AN3 | HOH 528 | $H_B$ 3.0 | HOH 528 | AO2' | $H_B$ 2.7 |
| | | | HOH 528 | HOH 103 | $H_B$ 2.9 |

**Supplementary Table 3 | Interactions of $NAD^+$ with NuoEF in the oxidized state.**

| First coordination sphere | | | Second coordination sphere | | |
|---|---|---|---|---|---|
| **Atom $NAD^+$** | **Residue Atom** | **Length [Å]** | **Residue Atom** | **Residue Atom** | **Length [Å]** |
| AO3' | Lys76$^F$ NZ | $H_B$ 2.9 | Lys76$^F$ N | Pro72$^F$ O | $H_B$ 3.2 |
| | | | Lys76$^F$ NZ | Val218$^F$ O | $H_B$ 2.9 |
| | | | Lys76$^F$ O | Ala80$^F$ N | $H_B$ 3.2 |
| | | | Lys76$^F$ NZ | FMN O2P | $H_B$ 3.2 |
| AO2' | Glu185$^F$ OE1 | $H_B$ 3.3 | Glu185$^F$ OE2 | Lys202$^F$ NZ | $H_B$ 2.9 |
| AO2' | Glu185$^F$ OE2 | $H_B$ 2.6 | Glu185$^F$ O | Ile189$^F$ N | $H_B$ 3.1 |
| AO3' | Glu185$^F$ OE1 | $H_B$ 2.6 | Glu185$^F$ N | FMN O2' | $H_B$ 3.0 |
| | | | Glu185$^F$ OE1 | HOH 308 (103) | $H_B$ 3.4 |
| AO2' | HOH 483 (528) | $H_B$ 2.4 | HOH 483 (528) | HOH 308 (103) | $H_B$ 2.8 |
| NO2' | HOH 648 | $H_B$ 2.8 | HOH 648 | Glu97$^F$ OE1 | $H_B$ 3.0 |
| NO2' | Gly68$^F$ O | $H_B$ 3.3 | Gly68$^F$ N | HOH 129 | $H_B$ 2.7 |
| NO3' | Gly68$^F$ O | $H_B$ 3.4 | | | |
| NO7 | Glu95$^F$ O | $E_R$ 2.9 | Glu95$^F$ O | HOH 150 (6) | $H_B$ 2.9 |
| | | | Glu95$^F$ N | HOH 79 (527) | $H_B$ 3.1 |
| | | | Glu95$^F$ OE2 | Asp103$^F$ N | $H_B$ 2.8 |
| | | | Glu95$^F$ OE1 | Arg104$^F$ N | $H_B$ 3.2 |
| | | | Glu95$^F$ OE1 | Tyr138$^F$ OH | $H_B$ 2.7 |
| | | | Glu95$^F$ OE2 | HOH 61 (526) | $H_B$ 2.7 |
| NO7 | HOH 545 | $H_B$ 2.5 | HOH 545 | HOH 61 (526) | $H_B$ 2.8 |
| | | | HOH 545 | HOH 241 | $H_B$ 2.9 |
| NO1 | HOH 145 (534) | $H_B$ 2.5 | HOH 145 (534) | Glu184$^F$ OE2 | $H_B$ 3.0 |
| | | | HOH 483 (528) | Gly394$^F$ N | $H_B$ 2.9 |
| NO2 | FMN O2' | $H_B$ 2.8 | | | |
| NO2 | FMN O3' | $H_B$ 2.6 | | | |
| AN3 | HOH 483 (528) | $H_B$ 2.5 | | | |

**Supplementary Table 4 | Preparation of the *A. aeolicus* G129S$^E$ and G129D$^E$ and the *E. coli* G135S$^E$ and G135D$^E$ variants.**

15 g (wet weight) Rosetta/pETBlue-1*nuoEF*$_{his}$ *nuoE* G129S cells were used.

| Preparation | Volume | Protein | NADH/ferricyanide-oxidoreductase activity total | NADH/ferricyanide-oxidoreductase activity specific | Yield |
|---|---|---|---|---|---|
| | [mL] | [mg] | [µmol·min$^{-1}$] | [µmol·min$^{-1}$·mg$^{-1}$] | [%] |
| Cytosol | 88 | 1074 | 3846 | 3.6 | 100 |
| Probond Ni$^{2+}$-IDA | 22.5 | 180 | 1706 | 9.5 | 44 |
| Superdex 200 16/60 | 24 | 90 | 1064 | 11.8 | 28 |
| SQ-15 | 5 | 29 | 538 | 20.1 | 14 |
| Superdex 200 16/60 | 2 | 22 | 466 | 21.2 | 12 |

10 g (wet weight) Rosetta/pETBlue-1*nuoEF*$_{his}$ *nuoE* G129D cells were used.

| Preparation | Volume | Protein | NADH/ferricyanide-oxidoreductase activity total | NADH/ferricyanide-oxidoreductase activity specific | Yield |
|---|---|---|---|---|---|
| | [mL] | [mg] | [µmol·min$^{-1}$] | [µmol·min$^{-1}$·mg$^{-1}$] | [%] |
| Cytosol | 62 | 742 | 2283 | 3.1 | 100 |
| Probond Ni$^{2+}$-IDA | 21 | 110 | 1105 | 10.0 | 48 |
| Superdex 200 16/60 | 10 | 40 | 567 | 14.2 | 25 |
| SQ-15 | 5 | 21 | 401 | 19.1 | 18 |
| Superdex 200 16/60 | 1.2 | 13.5 | 292 | 21.6 | 13 |

31 g (wet weight) BW25113Δ*nuo ndh::nptI*/pBAD*nuo nuoF$_{his}$nuoE* G135S cells were used.

| Preparation | Volume | Protein | NADH/ferricyanide-oxidoreductase activity | | Yield |
|---|---|---|---|---|---|
| | | | total | specific | |
| | [mL] | [mg] | [μmol·min$^{-1}$] | [μmol·min$^{-1}$·mg$^{-1}$] | [%] |
| Membrane | 19.0 | 1210 | 1551 | 1.3 | 100 |
| Detergentextract | 58.0 | 913 | 1272 | 1.4 | 82 |
| Fractogel EMD | 60.0 | 210 | 612 | 2.9 | 39 |
| ProBond $Ni^{2+}$-IDA | 0.8 | 4.3 | 202 | 47.0 | 13 |

42 g (wet weight) BW25113Δ*nuo ndh::nptI*/pBAD*nuo nuoF$_{his}$nuoE* G135D cells were used.

| Preparation | Volume | Protein | NADH/ferricyanide-oxidoreductase activity | | Yield |
|---|---|---|---|---|---|
| | | | total | specific | |
| | [mL] | [mg] | [μmol·min$^{-1}$] | [μmol·min$^{-1}$·mg$^{-1}$] | [%] |
| Membrane | 28.7 | 1828 | 2011 | 1.1 | 100 |
| Detergentextract | 125.0 | 1409 | 1690 | 1.2 | 84 |
| Fractogel EMD | 60.0 | 358 | 718 | 2.0 | 36 |
| ProBond $Ni^{2+}$-IDA | 0.7 | 7.0 | 315 | 45.0 | 16 |

**Supplementary Table 5 | Mutagenic primer to construct the *A. aeolicus* G129S$^E$, G129D$^E$ and S96M$^F$ and the *E. coli* G135S$^E$ and G135D$^E$ variants.**

| Oligonucleotide | Sequence | New restriction site |
|---|---|---|
| *nuoE* G135S_fwd | 5'-CAACTTGCTGC*TTA***AGT**AACTGTGAT AAAG-3' | AflII |
| *nuoE* G135S_rev | 5'-CTTTATCACAGTT**ACT***TAA*GCAGCAA GTTG-3' | AflII |
| *nuoE* G135D_fwd | 5'-CTTGCTGCCTG**GAC**AACTGTGATAA AGGGCCCAACATGATGATC-3' | ApaI |
| *nuoE* G135D_rev | 5'-GATCATCATGTTGGGCCCTTTATCAC AGTT**GTC**CAGGCAGCAAG-3' | ApaI |
| *nuoE*$_{axa}$G129S_fwd | 5'-CGTTCAGTGTCTG**AGC**GCCTGC*AGT* GAAGCTCCCGTG-3' | PstI |
| *nuoE*$_{axa}$G129S_rev | 5'-CACGGGAGCTTC*ACT*GCAGGC**GCT**C AGACACTGAACG-3' | PstI |
| *nuoE*$_{axa}$G129D_fwd | 5'-CGTTCAGTGTCTG**GAT**GCCTGC*AGT* GAAGCTCCCGTG-3' | PstI |
| *nuoE*$_{axa}$G129D_rev | 5'-CACGGGAGCTTC*ACT*GCAGGC**ATC**C AGACACTGAACG-3' | PstI |
| *nuoF*$_{axa}$S96M_fwd | 5´-GCAACGCGGACGAG**ATG**GAACCGG GTACCTTTAAGGACAGG | KpnI |
| *nuoF*$_{axa}$S96M_rev | 5´- CCTGTCCTTAAAGGTACCCGGTTC**C** **AT**CTCGTCCGCGTTGC | KpnI |

**Supplementary Note**

**Binding of $NAD^+$ and NADH to *A. aeolicus* NuoEF**

In the presence of 10 mM $NAD^+$ an extra electron density feature appeared that was modeled accordingly (**Fig. 2**). The electron density of the adenosine-ribose moiety was clearly visible while that of the nicotinamide was only weakly defined. In our model and in that described by Berrisford and Sazanov[17] the nicotinamide and the isoalloxazine ring of FMN interact by π-stacking in a distance of about 3.0 Å. The nicotinamide N7 forms hydrogen bonds with the backbone amides of $E97^F$ and $S96^F$. The side chain of $E97^F$ tilts upon NADH binding and forms a hydrogen bond with the hydroxyl-group of $Y180^F$ to arrange the nicotinamide in an optimal position for hydride transfer. NO2 of the nicotinamide is hydrogen bonded to the ribityl O2' and O3' of the FMN. The adenine ring is stabilized by π-stacking interactions with the conserved $F71^F$ ($F70^1$ in *T. t.*) and the variable $Y205^F$ ($F205^1$ in *T. t.*). The variable $F79^F$ does not interact with the adenine ring while a stacking interaction of this position ($F78^1$ in *T. t.*) with the adenine moiety was described by Berrisford and Sazanov[17]. The conserved $E185^F$ formed two hydrogen bonds to adenosyl-ribose, as described for other dehydrogenases[46]. Common to both structures the loop formed by residues 201–206 moves 0.9 Å towards the binding site upon NADH binding. Accordingly, the side chain of $Y205^F$ re-orientates such that it exerts stacking interactions with the adenine. The glycine-rich loop of complex I (residues 65–70) moves 0.6–1.3 Å apart opening up the binding site, assisted by a movement of the preceding α-helix by about 0.5 Å. In both structures, no hydrogen bonds between the glycine-rich loop and the pyrophosphate occur. The carbonyl group of $G67^F$ ($G66^1$ in *T. t.*) forms a hydrogen bond to the adenine N7. However, the interaction of $G68^F$ ($G67^1$ in *T. t.*) and N71 reported for the *T. thermophilus* complex is not present in the *A. aeolicus* enzyme as well as the described interaction of $G67^F$ and $G68^F$ with NO3' of the nicotinamide ribose[17]. In contrast, an interaction of $G68^F$ ($G67^1$ in *T. t.*) with NO3' of $NAD^+$ is observed in the oxidized NuoEF. In both redox states the backbone amide of $G67^F$ ($G66^1$ in *T. t.*) is hydrogen bonded to the O1P of the FMN. In addition, $K76^F$ ($K75^1$ in *T. t.*) forms a hydrogen bond with the FMN O2P. Upon NADH binding, $K76^F$ does not lose this bond as described[17], but it forms an additional bond to the AO3' of the adenosine ribose. The described interaction of $K76^F$ with FMN O5' was not detected. In contrast to the *T. thermophilus* structure[16], $K202^F$ is hydrogen-bonded to the carboxylate of $E184^F$ and the carbonyls of $R200^F$ and $I392^F$. In the oxidized NuoEF a hydrogen bond is formed by $K202^F$ and $E185^F$. The backbone amide of $E185^F$ also forms a hydrogen bond with the FMN O2'. This bond is not contained within the *T. thermophilus* structure. This difference may be caused by the different orientation of the ribityl moiety of FMN in both structures (see below). $E185^F$ ($E185^1$ in *T. t.*) makes two strong hydrogen bonds with the AO2' and AO3' of the adenosine ribose. However, as the nicotinamide moiety of

NAD$^+$ is not stringently defined in the electron density maps, and the position of the vicinal ribose sugar indicates that it points away from the actual binding pocket, unable to stack onto the isoalloxazine system of FMN.  In the model, the ligand was consequently only included as ADP-ribose. The nicotinamide moiety was modeled according to the positions with a defined electron density. We rationalize the observed ejection of nicotinamide from the binding pocket with the observed peptide flip at residue G95$^F$.

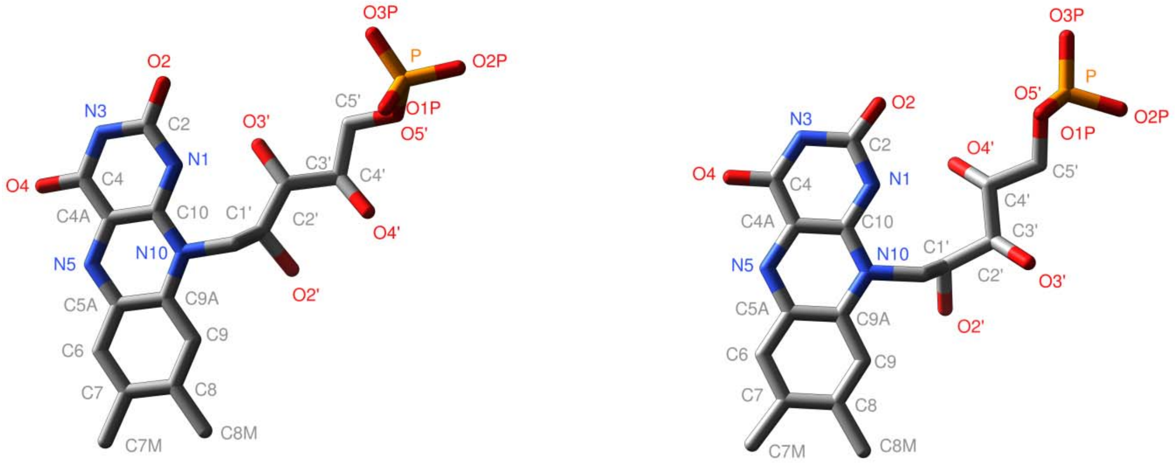


Structural model of the FMN cofactor in *T. thermophilus* (left; pdb-entry: 3IAM16) and *A. aeolicus* (right) complex I (this work).

46. Lesk, A.M. NAD-binding domains of dehydrogenases. *Curr. Biol.* **5**, 775-783 (1995).
